# Exploring patient experiences of illness and diagnostic pathways in self-reported Q fever cases

**DOI:** 10.3389/fpubh.2025.1701818

**Published:** 2026-01-27

**Authors:** Tabita Tan, Jane Heller, Lynne Hayes, Anke Wiethoelter

**Affiliations:** 1 Faculty of Science and Health, Charles Sturt University, Wagga Wagga, NSW, Australia; 2 Faculty of Science, The University of Melbourne, Parkville, VIC, Australia

**Keywords:** *Coxiella burnetii*, health care, medical diagnosis, patient experience, Q fever, qualitative analysis

## Abstract

**Introduction:**

Q fever is an underestimated zoonotic disease with serious human health implications. This study explores patient experiences to characterize the impact of illness on quality of life, the pathway to diagnosis, and satisfaction with healthcare.

**Methods:**

An online survey was conducted to gather self-reported cases of Q fever, and follow-up interviews were carried out with consenting survey participants to represent a range of experiences related to diagnosis and illness severity. Quantitative data were analyzed descriptively, and thematic analysis was performed on qualitative data.

**Results:**

Quality of life was severely affected in 74% of patients, with many requiring hospitalization (48%) and taking time off work or school (87%). While some participants reported being unwell for over 10 years, the majority seem to recover within 6 months. Diagnosis was delayed for several participants, many of whom had multiple medical visits (>10) and were ill for years before receiving a medical diagnosis. The majority of diagnoses were made by general practitioners, whose knowledge of Q fever was an important factor in achieving timely diagnosis. The majority of participants reported being exposed to livestock around the time they likely contracted the illness. Six main themes were derived from the interviews: experience of physical illness, impact on life, emotional experience, managing and surviving Q fever, experience with the medical process, and importance of awareness.

**Conclusion:**

Q fever has the potential to cause severe and life-changing consequences. Obtaining a timely diagnosis can be difficult, and many patients reported dissatisfaction with the quality of their healthcare. Adopting a model of patient-centered care and increasing awareness of Q fever could improve health outcomes and provide better support for affected patients.

## 1 Introduction

Q fever is a serious zoonotic disease caused by the bacterium *Coxiella burnetii*. Infection is commonly acquired through contact with infected cattle, sheep, and goats; therefore, individuals who work closely with livestock or live in rural areas are often perceived to be at the greatest risk (1). *Coxiella burnetii* can persist for long periods in the environment and is highly infectious; as a result, individuals can also acquire the infection from contaminated environments or materials in the absence of animal contact (2, 3). Although the majority of human infections result in asymptomatic seroconversion, some individuals may experience symptoms ranging from acute, self-limiting influenza-like illness to severe clinical cases and chronic infections that require hospitalization and extensive treatment (4). These varying syndromes associated with Q fever have been characterized, although definitions are not uniform. Many individuals with chronic Q fever or Q fever fatigue syndrome continue to face health problems for up to 10 years after the initial infection (5).

Achieving a timely and definitive diagnosis is challenging due to the clinical polymorphism of *C. burnetii* infections, and complicated laboratory testing is necessary to confirm suspicions of infection (1, 6). Diagnosis relies on clinicians' knowledge and suspicion of Q fever, requiring a work-up that includes obtaining a detailed history, identifying epidemiological risk factors, performing specific examinations, and conducting initial screening investigations that involves ordering the correct diagnostic tests (1, 4). Treatment of symptomatic patients at an early stage with a course of doxycycline is effective and can prevent serious complications or progression. Due to the potential for severe and chronic sequelae in a small number of infected individuals, it is recommended that empirical antibiotic therapy be initiated as soon as the presentation and clinical history suggest a zoonotic disease (1, 4, 5, 7).

Australia has one of the highest rates of Q fever notifications globally, with 500 to 600 cases (notification rate of 2.1 cases per 100,000 people per year) reported annually. The majority of these cases occur among residents of Queensland and New South Wales (7–10). While the most frequent occupations among notified Q fever cases involve contact with livestock, many cases have no known occupational risks or reported animal exposure pathways (3, 9, 11). Seroprevalence studies in Australia indicate that Q fever infections occur across both rural and metropolitan populations at notable rates (8, 12). The expected number of clinical Q fever cases, based on seroprevalence studies, far exceeds the reported cases, highlighting a significant issue of underdiagnosis and the broader underrecognition of Q fever as a public health issue in Australia (8, 10). The highly effective Q fever vaccine, Q-VAX^®^, is approved for human use in Australia and has been used successfully in vaccination campaigns to reduce the incidence of the disease in some high-risk occupational groups. However, vaccine coverage remains incomplete, and the reported cases in Australia continue to be high (9, 11).

Studies examining the consequences of Q fever in patients have primarily focused on clinical outcomes and treatments, with limited research on self-perceived health status and quality of life. However, self-perception of health is important for the wellbeing of the patient. Additionally, little is known about patients' experiences with illness, their diagnostic journey, and satisfaction with care. Therefore, the objective of this study is to provide an exploratory account of Q fever patient experiences based on self-reported data. Specifically, we aim to (1) characterize the impact of Q fever illness on patients, symptoms, duration, and health-related quality of life and (2) examine the diagnostic pathway and satisfaction with the healthcare provided. Recognizing the impact of Q fever on health-related quality of life and identifying the potential issues patients face during the diagnostic process can help improve patient outcomes and address public health challenges.

## 2 Methods

### 2.1 Research design and data collection

This study targeted adults and guardians of children who self-reported experiencing Q fever in Australia and was conducted in two parts: a survey and follow-up interviews. The survey was advertised through Q fever Facebook groups, websites, local country newspapers, and online regional radio channels. It was conducted from October 2021 to November 2022. The survey was administered as an online questionnaire via the Survey Monkey^®^ platform, including both closed- and open-ended questions. It covered four sections: (1) experience of illness, (2) experience with the diagnostic process, (3) Q fever acquisition, and (4) feelings about the diagnostic journey. Participants could complete the survey either as adults responding for themselves or as guardians answering on behalf of a child. Medical data on diagnosis, healthcare, and impacts were self-reported, relying on the respondents' recall and disclosure. No clinical or laboratory confirmation was collected. Skip logic was applied to customize the survey based on participants' self-reported method of diagnosis, which included diagnosis by a medical practitioner, a positive pre-screening test for Q fever vaccination, and self-diagnosis. The survey question on quality of life encompassed impact categories specifically adapted from the domains outlined by Forestier et al. (13), including physical, psychological, social, and functional aspects. Respondents could provide contact details for a follow-up interview at the conclusion of the survey. Participants for the follow-up interview were selected through purposive sampling from the list of consenting participants to represent different methods of diagnoses (self-diagnosis, vaccination pre-screening, and medical diagnosis) and illness severity (asymptomatic, acute, and chronic cases). Only adults were selected for interviews, which were conducted over the phone or via Zoom (14). Participants provided verbal consent prior to commencing the interview and were then asked to: (1) talk about themselves and why they decided to participate in the interview; (2) describe their experiences with Q fever, including symptoms, duration, and its impact on their life; and (3) explain how their Q fever was diagnosed, including thoughts and feeling about the diagnostic process. TT and LH conducted the interviews.

### 2.2 Data analysis

Survey data were imported into R (15) for descriptive and graphical analysis. For days in hospital, days off work or school, and days to diagnosis, data were grouped into quartiles. Patient data were stratified into two groups: Queensland/New South Wales and all other states, as QLD and NSW have a high incidence of Q fever. Differences between the groups were tested using the Wilcoxon rank-sum test for continuous variables, and only statistically significant results are reported. Cronbach's alpha was calculated for each Likert scale to assess internal consistency, using the alpha() function from the psych package. Open-ended survey responses were imported into NVIVO (16) and coded for recurring ideas. Interviews were audio-recorded and transcribed verbatim by a professional transcription company. Reflexive thematic analysis (17, 18) was used to analyse and derive themes from the data. All audio recordings were reviewed alongside interview transcripts by TT for data familiarization, and codes were generated inductively. Initial codes were cross-checked by AW, iteratively adjusted by TT, and used to code the transcripts in NVIVO 12 (16). Coded data were evaluated for unifying themes and for relationships between characterizing themes.

## 3 Results

### 3.1 Survey

Survey data were obtained from 191 participants, including 171 adults (21 years and older) and 20 carers/guardians of children under 20 years. Demographics and experiences of illness are detailed in Table 1. Furthermore, 40% of the respondents identified as female and 55% as male.

**Table 1:** Demographics and illness experiences among the 191 adults and children affected by Q fever in Australia.

**Characteristic**	Number of respondents^*^(%)
**Sex**	
Male	108 (57)
Female	81 (42)
Prefer not to say	2 (1)
**Age**	
0–4	4 (2)
5–9	6 (3)
10–14	6 (3)
15–20	4 (2)
21–40	36 (19)
41–60	70 (37)
>60	62 (33)
**Clinical signs**	
Fatigue	168 (88)
Fevers and chills	166 (87)
Sweats	160 (84)
Muscle and joint pain	158 (83)
Headaches	153 (80)
Weight loss	110 (58)
Coughing	94 (49)
Nausea/vomiting/diarrhea	90 (47)
Stomach pain	88 (46)
Pneumonia	42 (22)
Endocarditis	36 (19)
Hepatitis	34 (18)
**Time to recovery**	
1–3 weeks	18 (23)
1–6 months	25 (31)
7 months−2 years	12 (15)
3–10 years	11 (14)
11–>15 years	3 (4)
Not fully recovered	11 (14)
**Hospitalized**	
Yes	75 (48)
No	80 (52)
**Time off work or school**	
Yes	139 (87)
No	20 (13)
**No. of days off work or school**	
1–75 days	90 (76)
76–150 days	16 (13)
151–225 days	6 (5)
226–300 days	7 (6)
**Awareness of Q fever**	
**Awareness of Q fever before diagnosis**	
Yes	93 (51)
No	90 (49)
**Suspicion of Q fever among those who were aware before**	
**diagnosis**	
Yes	43 (51)
No	41 (49)

^*^Most variables had up to 18% missing data, resulting in N < 191, except for “Sex,” which was answered by all 191 respondents. “Time to recovery” and “Days off work or school” had large amounts of missing data at 58 and 38%, respectively. The high volume of missing data may be attributed to the difficulty the respondents faced in recalling these continuous variables.

Fatigue, fevers and chills, sweats, muscle and joint pain, and headaches were the predominant symptoms reported by the respondents. The median recovery time corresponded to the 1–6 month category, the median number of days in hospital was 7 (range 1–120), and the median time off work or school was 25 (range 0–over 300) days.

**Figure 1:**
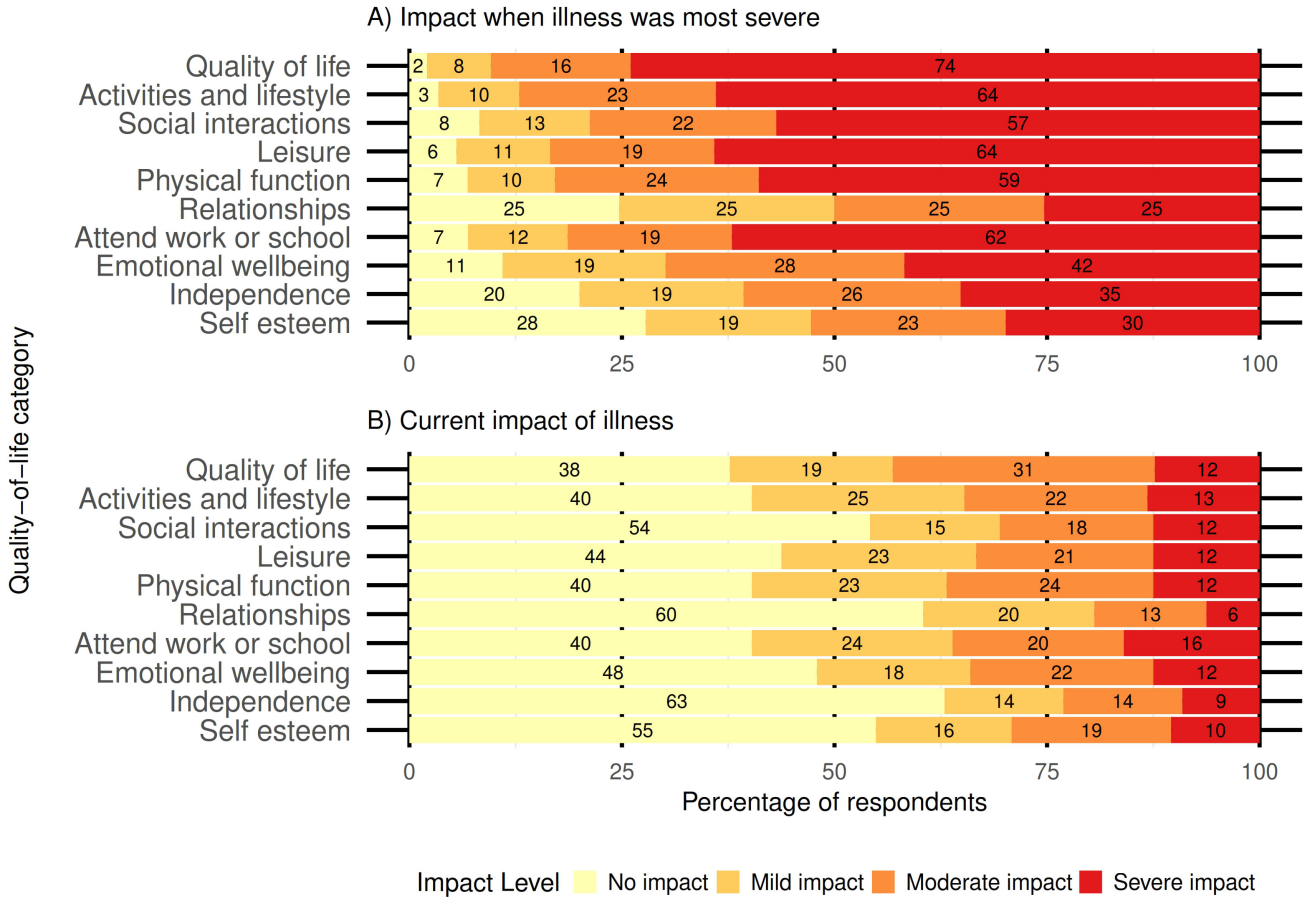
Likert scale showing the severity of impact of Q fever among adults and guardians of children in Australia: **(A)** at the time when the illness was most severe and **(B)** current impact of the illness.

The perceived impact on overall quality of life and several other dimensions was severe for most patients at the peak of illness, with a shift toward improvement at the current time, as an increased proportion of respondents reported no impact on overall quality of life or any other dimensions (Figure 1). Reliability analysis indicated high internal consistency for both 10-item scales (α = 0.94 when illness was most severe, α = 0.98 for current impacts).

Some patients reported self-diagnosis based on symptoms (6%) or were positive on pre-screening for vaccination (22%). Most patients (72%) were diagnosed by a medical practitioner, with a median of two doctor visits prior to medical diagnosis, predominantly in small regional towns and large regional centers (Table 2). The patients in Queensland and New South Wales had significantly fewer doctor visits prior to diagnosis (median = 2) compared to those in other states (median = 3; *W* = 508, *p* = 0.023). The median time to medical diagnosis was 14 days for adults and 28 days for children. Queensland and New South Wales had a significantly shorter time to medical diagnosis (median = 12) compared to other states (median = 35; *W* = 555, *p* = 0.003). Most adult patients who received a medical diagnosis first attended general practitioners (60%) and were diagnosed by general practitioners (50%), but most children were diagnosed in a hospital setting (54%). Among participants with other cases related to theirs, 60% (15/25) reported that this influenced their medical diagnosis.

**Table 2:** Characteristics of experiences among the 191 adults and children affected by Q fever in Australia, including a diagnostic pathway and self-reported likely exposure for Q fever acquisition.

**Characteristics**	**Number of respondents** ^*^ **(%)**	
**Method of diagnosis**		
Self-diagnosed	10 (6)	
Pre-screening for vaccination	36 (22)	
Medical practitioner	121 (72)	
**Medical facility where the diagnosis was made**		
Remote (>100 km from nearest town)	1 (1)	
Rural village or locality (< 1,000 pp)	6 (5)	
Small regional town (1,000–20,000)	55 (48)	
Large regional center (>20, 000)	29 (25)	
Metropolitan—capital cities	24 (21)	
**State in which the diagnosis was made**		
NSW	50 (43)	
QLD	39 (34)	
VIC	10 (9)	
SA	11 (10)	
NT, TAS and WA	5 (4)	
**Time to diagnosis by medical**	**Adult**	**Child**
**practitioner**		
1–10 days	43 (43)	4 (40)
14–46 days	35 (35)	3 (30)
61–304 days	12 (12)	1 (10)
335–5,475 days	11 (11)	2 (20)
**Medical facilities attended for those who received a medical**		
**diagnosis**		
**First medical facility attended when**	**Adult**	**Child**
**ill**		
Emergency department/hospital	25 (24)	5 (38)
General practitioner	62 (60)	5 (38)
Medical center	7 (7)	3 (23)
Ambulance	3 (3)	
Other	6 (6)	
**Medical facility where the diagnosis was made**		
Emergency department	10 (10)	3 (23)
Hospital	30 (29)	7 (54)
General practitioner	51 (50)	2 (15)
Medical center	5 (5)	1 (8)
Other	7 (7)	
**Medical diagnosis: other cases related**		
Yes	35 (31)	
No	68 (61)	
Don't know	9 (8)	
Cattle	96 (68)	12 (75)
Sheep	73 (51)	6 (38)
Goats	42 (30)	7 (44)
Horses	37 (26)	5 (31)
Pigs	23 (16)	3 (19)
Cats	39 (27)	5 (31)
Dogs	72 (51)	7 (44)
Native wildlife	50 (35)	5 (31)
Untreated animal products or waste	57 (40)	5 (31)

^*^Variables “method of diagnosis” and “reported animal exposure” had up to 16% missing data, resulting in N < 191. Other variables had up to 7.5% missing data, resulting in N < 121, which is the number of respondents whose diagnosis was made by a medical practitioner.

From open-ended survey responses, the respondents described issues with the diagnostic process, such as delays in diagnosis (*n* = 22) and initial misdiagnosis (*n* = 26) with other diseases (e.g., cancer, meningitis, leukemia, and common viral illnesses). Q fever osteomyelitis in the children was initially misdiagnosed as arthritis, sprains, or fractures (*n* = 4). The respondents commented that the doctor's prior experience, knowledge of presenting symptoms, awareness of infection risk factors, and ability to obtain a relevant history (*n* = 13), as well as the patient's knowledge of risk factors and illness to request Q fever testing (*n* = 20), had an influence on timely diagnosis. A total of 19 (10%) respondents felt their doctor lacked knowledge of Q fever, and 22 (12%) experienced a lack of concern, insufficient follow-up, or difficulty convincing their doctor to test for Q fever. Furthermore, nine (5%) respondents appreciated their prompt diagnosis and medical care.

All children and most adult respondents (125/142, 88%) reported exposure to cattle, sheep, or goats around the time of likely infection (Table 2). Direct and indirect contact with livestock, wildlife, and ticks were described in open-ended survey responses as possible routes of acquisition. Most respondents reported being livestock workers or living on farms or in rural areas (*n* = 116). Other responses included wildlife carers (*n* = 1); mowing contaminated ground (*n* = 2); visiting livestock facilities as contractors, for research, work experience, or tourism (*n* = 8); hiking/camping in parks (*n* = 3); and visiting petting zoos (*n* = 2). In some cases (*n* = 17), there was no direct animal contact, but infection was suspected to result from nearby livestock/wildlife (*n* = 9), contaminated clothing (*n* = 2), dust storms (*n* = 2), or passing stock trucks/abattoirs (*n* = 6).

Most respondents (59%) felt that the pathway to medical diagnosis was not straightforward but were satisfied with the medical investigation (57%) and their diagnosis of Q fever (88%). Satisfaction with their Q fever diagnosis indicates that the respondents were confident in its accuracy, felt it led to effective treatment, and were reassured by a clear explanation for their symptoms, valuing the doctor's competence and care.

### 3.2 Interviews

A total of 13 interviews were conducted, lasting an average of 37 min (range: 15–72 min). The participants were aged 41 to over 70 years old, identified as male (*n*= 11) or female (*n*= 2), and had experienced different forms of Q fever (one asymptomatic, five acute, and seven chronic cases). Diagnostic methods varied among the participants: 10 received a medical diagnosis, substantiated by details shared during the interview; two self-diagnosed based on their symptoms; and one was identified as having Q fever during pre-screening for vaccination. Support persons participated in two interviews at the patient's request.

A total of six main themes were derived from the interviews: (1) experience of physical illness, (2) impact on life, (3) emotional experience, (4) managing and surviving Q fever, (5) experience with the medical process, and (6) importance of awareness. These themes largely reflect the challenges faced by individuals experiencing severe or chronic symptoms and the difficulties encountered when the pathway to diagnosis and treatment is complex. The patients described suddenly becoming very ill and incapacitated despite being previously fit and healthy (theme 1). Those with severe chronic illness struggled to return to work, socialize, and perform daily activities, describing it like a hidden disability that was misunderstood or dismissed by others (theme 2). This impact on their lives resulted in feelings of isolation, anxiety, sadness, vulnerability, depression, and low self-esteem, to the point where some of them wanted to die (theme 3). The patients discussed the importance of support from family and friends and shared experiences with other patients. Many reported having mostly recovered and learned to manage residual symptoms by recognizing triggers or adjusting their mindset (theme 4). Some patients expressed appreciation for their doctor's knowledge and for the rapid assessment and response to their illness. However, most respondents reported that it was difficult to obtain a medical diagnosis of Q fever for their illness, and a few were troubled by what they perceived to be a lack of concern or follow-up from the medical system. These patients expressed a desire to be heard, respected, and acknowledged as capable of recognizing dysfunction in their own bodies (theme 5).

**Figure 2:**
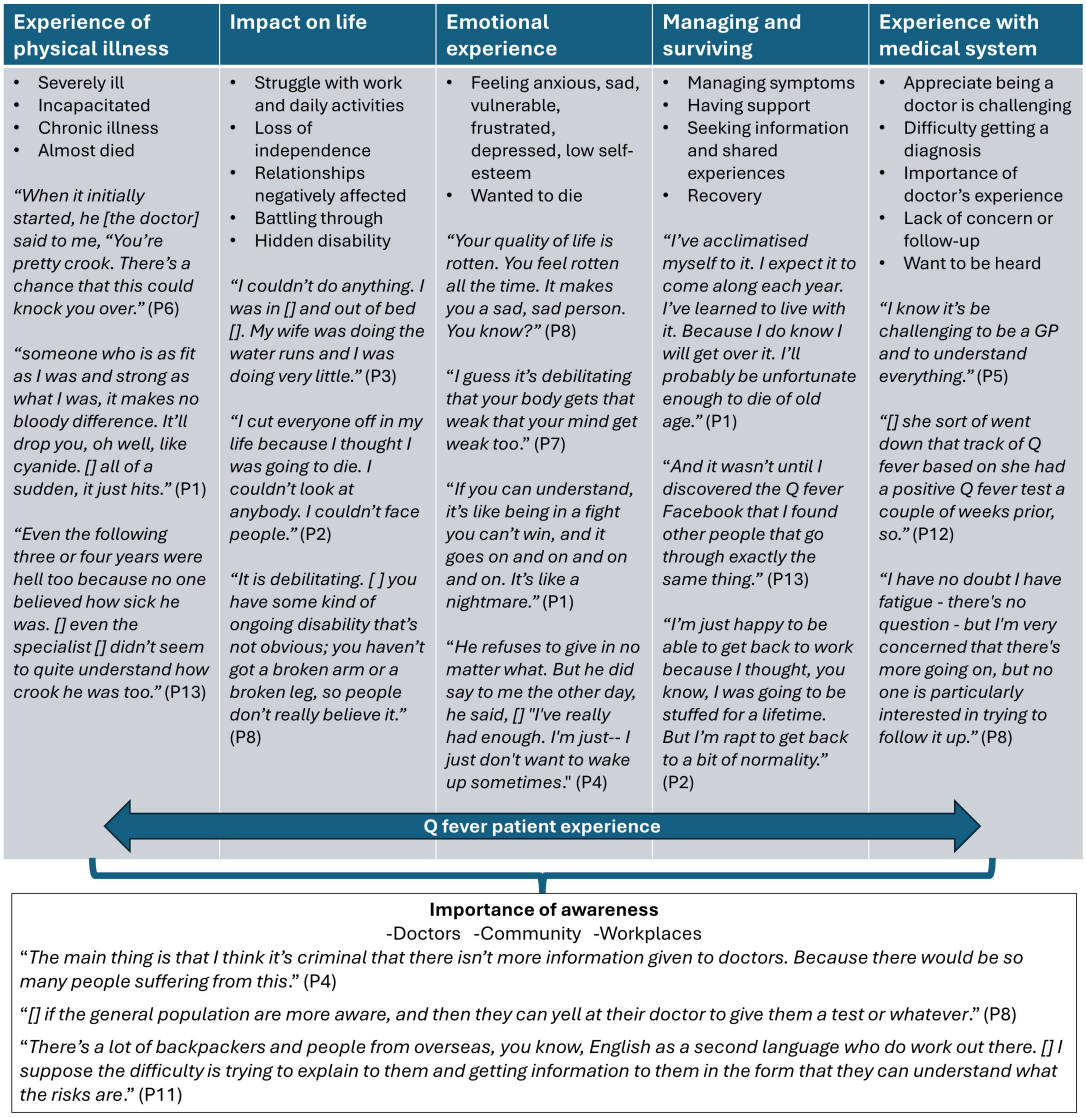
Concept map illustrating the relationships between the themes derived from the interviews with adults affected by Q fever in Australia. Each bullet point represents a code that characterizes the main content of the theme, and quotes from the interview respondents are included to illustrate each theme. All quotes have been de-identified and edited for clarity. Omissions or insertions are indicated by square brackets, but the original meaning of the quotes remains unchanged.

The relationship between themes is represented using a conceptual model (Figure 2). The first five themes are interconnected, forming the central facets that define the patient experience. The sixth theme, importance of awareness, represents an outcome of the Q fever experience. Helping others, by increasing awareness among doctors and the community, improving health outcomes, and reducing misunderstanding, was cited by the patients as a major reason for participating in this study.

## 4 Discussion

The results of this study are specific to patients with a self-reported Q fever diagnosis, who are likely to have experienced more severe symptoms or chronic sequelae. The findings indicate that Q fever can have a profoundly debilitating and life-changing impact on patients with long-term illness. Fatigue and other symptoms considerably hinder daily activities; disrupt work and social roles; and affect physical, mental, and emotional wellbeing. The high burden of disease, long-term health effects, and reduced quality of life associated with severe and chronic Q fever symptoms have also been documented in other studies (5, 19–22). It is comparable to other hidden disabilities, such as autoimmune diseases or mental health issues, where suffering is not physically apparent and others, including health providers, may not fully understand, assist, or even believe the patient's experiences. Consequently, patients can feel misunderstood or isolated and face challenges in obtaining the support and care that they need. Fortunately, severe impairment tends to subside, and most patients in this study reported a reduced impact of Q fever as they eventually recovered from their illness, learned to manage residual symptoms, and returned to normal daily activities.

In this study, most participants stated substantial recovery within 6 months, which is consistent with findings from another Australian study, where the duration of post-Q fever illness averaged ~7 months for individuals with high acute illness severity and 70 days for those with low severity (23). Similarly, Morroy (24) found that recovery from fatigue following acute Q fever in the Dutch outbreak occurred within 6–12 months for most patients. The relatively faster recovery observed in this study's cohort compared to the Dutch cohort may reflect differences in population characteristics, pathogen characteristics, healthcare access, and treatment practices.

The pathway to diagnosis was complex for many patients who experienced multiple medical visits and misdiagnoses before learning they had Q fever. Timely diagnosis was attributed to the doctor's knowledge and experience with the disease, as well as the patient's ability to recognize risk factors and symptoms, enabling them to request Q fever testing. This may explain the fewer doctor visits before diagnosis observed in New South Wales and Queensland, where Q fever incidence is highest. This study also highlights the important role of general practitioners in the early recognition of Q fever. Timely and accurate diagnosis enables effective treatment and recovery (25), and prior research shows that general practitioners' familiarity with Q fever decreases the risk of hospitalization (26). In contrast, specialist care was most important for diagnosing Q fever in children, suggesting that children are not commonly considered at risk for the disease. Children often present with clinical signs that are not widely recognized as consequences of Q fever, such as osteomyelitis, which can lead to delays in diagnosis (27, 28). Further studies specifically designed to compare adults and children would be valuable for understanding differences in diagnostic processes and for identifying opportunities to improve early recognition in pediatric cases.

Several patients were dissatisfied with their healthcare, feeling that their concerns were ignored or that their illness was underestimated and dismissed due to their doctors' limited knowledge of Q fever. Insufficient disease knowledge among healthcare workers, particularly regarding the long-term effects of Q fever, has been identified as a major barrier to satisfactory care for patients (29). Similarly, a Dutch study found that most chronic Q fever patients rated their care poorly, highlighting a disconnect between patient needs and the care provided (5). This may reflect a mismatch in perceptions of “positive health,” where physicians often focus on biomedical health, whereas patients consider physical, mental, functional, occupational, and social aspects as equally important to overall wellbeing (5). The participants in our study also perceived Q fever to negatively impact their quality of life across these domains. A high proportion of participants indicated that their quality of life was severely impacted by Q fever, compared to 50% in other studies of the Dutch Q fever outbreak (5, 30). This discrepancy may be due to the differences in participant recruitment. However, given Q fever's variability and its complex management needs, a holistic, patient-centered approach would be beneficial (5, 29, 31–33). For example, the participants described a shift in mindset as an important part of managing Q fever, and offering cognitive–behavioral therapy rather than prolonged antibiotic treatment has been found to be more beneficial for some individuals experiencing long-term illness (34). Patient experience is an important measure of health outcomes and the effectiveness of the healthcare system (31). Customizing care to an individual's values and presenting symptoms can improve satisfaction and health outcomes while reducing unnecessary diagnostic tests, specialist referrals, and hospitalisations (31, 35–37).

The lack of disease familiarity presents a significant barrier to diagnosis, as Q fever awareness and knowledge among medical practitioners and communities—even individuals at high risk—are generally low (2, 38). Difficulties in obtaining a timely and accurate diagnosis are further compounded by the clinical polymorphism of Q fever, complexity of diagnostic testing, constraints on clinician time and the increasing volume of biomedical and clinical data that clinicians have to assimilate (7, 25). Although some participants acknowledged the challenges faced by medical practitioners , the value of education and awareness around Q fever was unanimously expressed by the interviewees. Diagnostic delays were reported more frequently in low-prevalence states. Although the reasons for this observation cannot be determined from the data, it suggests potential differences in clinician awareness and diagnostic practices between states, which could be explored in future research. Updating medical curricula to include major zoonotic diseases, along with ongoing online training as part of professional development for general practitioners, is recommended. Participant testimonies highlight the severe and protracted impacts of Q fever, and their willingness to share suggests that patient stories could be incorporated into media or public education initiatives to engage the community and dispel misconceptions (39). Raising awareness among health professionals and the community, such as through continuing medical education and public health campaigns, is necessary to improve early detection, quality of care, and disease prevention through vaccination and infection control measures (29, 38). It also fosters greater understanding, empathy, and support for those experiencing severe and chronic symptoms of Q fever.

The high proportion of perceived exposure to livestock around the time of likely infection suggests that many patients were living and working in rural areas, supporting transmission via traditional routes of direct contact with cattle, sheep, and goats. Concerningly, non-traditional routes—including wildlife contact, occupational exposure beyond livestock handling, and environmental exposure—were also described. These non-traditional routes have been noted in numerous other studies to play a substantial role in human transmission (1, 2, 40–43). Acquisition via non-traditional routes can be problematic, as it is very likely that without a specific history of exposure to farm animals or a high-risk occupation such as abattoir workers, Q fever is not even considered as a differential diagnosis (41). There is evidence to suggest that *Coxiella burnetii* is more common in the environment than expected and that risk determinants for infection and concurrent perspectives as to when to consider Q fever as a possible differential diagnosis need to be broadened (1, 28, 40, 41, 44).

Controlling the source of human infection in Australia is challenging due to the absence of systematic surveillance or vaccination programs in livestock, as well as the potential role of native animals, such as kangaroos and other macropods, as reservoirs. This is further compounded by the largely asymptomatic nature of *Coxiella burnetii* infection in animals and the environmental persistence of the organism, which complicates source identification and management. In many situations, Q fever in animals is only identified retrospectively, after several associated human cases have already been diagnosed (2, 45), highlighting the difficulty of detecting infection at the animal and environmental level before spillover occurs. For these reasons, the primary control strategy in Australia revolves around protecting at-risk people through improved awareness and vaccination (40).

Although the characteristics of the sample population (sex, age, exposure patterns) broadly align with the known epidemiological profile of Q fever in Australia, where most notifications are among middle-aged male individuals with livestock exposure (1), the sample size is small compared to the number of potential infections in the country. Voluntary participation in this study, with recruitment through social media support groups, local radio, and newspapers, is likely biased toward patients experiencing more severe disease complications, who were most motivated to respond. This bias was particularly evident in the interviews, where the participants with more severe chronic disease and greater dissatisfaction with medical care tended to speak more extensively, contributing to the qualitative analysis emphasizing longer diagnostic delays and more challenging experiences. Therefore, the perspectives captured may over-represent severe cases rather than the full spectrum of Q fever experiences in Australia and should not be generalized to the majority of Q fever cases, which are asymptomatic seroconversions or involve mild illness (1, 20).

This study did not account for the potential confounding impact of comorbidities or the misdiagnosis of other conditions as Q fever due to the absence of clinical data. Conditions with overlapping symptoms, such as depression or chronic fatigue syndrome, were not explicitly ruled out or reported, potentially leading to misclassification bias. Including detailed questions to differentiate these factors might have added complexity, risking participant confusion or survey fatigue. In addition, self-reported data rely on participant recall and presumed Q fever diagnosis without confirmation, which may compromise the accuracy of the findings. Future research with clinically confirmed cases that distinguishes symptoms specifically attributable to Q fever would be valuable. Most participants were from Queensland and New South Wales, reflecting the higher prevalence of Q fever in these states and the recruitment strategies used, while experiences from low-prevalence states such as Victoria and South Australia are likely underrepresented. Consequently, our findings may not fully capture regional differences, and any comparison between states should be considered indicative rather than confirmatory.

Despite these shortcomings, this study extends the existing literature on the lived experience of Q fever patients and brings attention to the significant long-term health impact that this disease can have on individuals. Most of the patients reported having a medical diagnosis of Q fever. While self-report can carry the risk of recall bias, Q fever is generally diagnosed through laboratory testing, making deliberate misreporting less likely. Moreover, the patterns observed in this study are consistent with the known clinical variability of Q fever and align with findings from other studies on the Dutch outbreak that support the importance of patient-centered care. Self-reported data remain a valuable tool for capturing patient experiences, particularly in conditions such as Q fever, where long-term effects can be difficult to assess through clinical records alone. Q fever is vastly underdiagnosed in Australia (8, 10), and including self-diagnosed patients, those who suspected they had Q fever but did not receive a formal medical diagnosis, offers valuable insights into barriers to diagnosis. Self-diagnosis reflects real-world behaviors and community perceptions of Q fever, which are critical for understanding its broader public health implications. These individuals had carefully evaluated their symptoms against available information and reported experiences similar to those with a confirmed diagnosis, suggesting that self-diagnosed cases may represent an underrecognised yet clinically relevant group. Furthermore, much of the patient experience was explored qualitatively, and data saturation was reached when no new insights were gained after 11 interviews, which is consistent with evidence that 6–12 interviews are sufficient in studies with focused objectives and relatively homogenous populations (46, 47).

## 5 Conclusion

Severe and chronic symptoms of Q fever, while not physically obvious, can significantly impact quality of life and lead to patient dissatisfaction with the standard of their healthcare due to the multifaceted nature of the disease. Delays in diagnosis may occur due to limited clinician familiarity or a lack of direct exposure to livestock. This underscores the need to raise awareness among primary healthcare practitioners and communities beyond the agricultural sector and to implement patient-centered care models that acknowledge and support patients' individual values, circumstances, and contributions to their own healthcare.

## Data Availability

The datasets presented in this article are not readily available because of the confidential nature of the participants' involvement in the project. Requests to access the datasets should be directed to ttan@csu.edu.au.
